# Using the Delphi Technique to Determine Which Outcomes to Measure in Clinical Trials: Recommendations for the Future Based on a Systematic Review of Existing Studies

**DOI:** 10.1371/journal.pmed.1000393

**Published:** 2011-01-25

**Authors:** Ian P. Sinha, Rosalind L. Smyth, Paula R. Williamson

**Affiliations:** 1 University of Liverpool, Alder Hey Children's Hospital, Liverpool, United Kingdom; 2 Department of Biostatistics, Faculty of Medicine, University of Liverpool, Liverpool, United Kingdom

## Abstract

Ian Sinha and colleagues advise that when using the Delphi process to develop core outcome sets for clinical trials, patients and clinicians be involved, researchers and facilitators avoid imposing their views on participants, and attrition of participants be minimized.

**Summary Points**

- Studies that use the Delphi process for gaining consensus around a core outcome set for clinical trials should be of sufficiently high quality in order for their recommendations to be considered valid.
- We report a systematic review of 15 studies that used the Delphi technique for this purpose, in which we identified variability in methodology and reporting.
- To improve the quality of studies that use the Delphi process for developing core outcome sets, we recommend that patients and clinicians be involved, researchers and facilitators avoid imposing their views on participants, and attrition of participants be minimised.
- Methodological decisions should be clearly described in the main publication in order to enable appraisal of the study.

## What Are Core Outcome Sets and Why Are They Useful?

Good clinical trial design requires researchers to specify in advance, in the protocol, those outcomes to be measured. If research has not been conducted to identify the most appropriate clinical trial outcomes in a given condition, three problems may impair the usefulness of the research in informing clinical practice. Firstly, researchers can select outcomes that suit their needs, at the expense of outcomes that are of most importance to patients or clinicians [1]–[3]. Secondly, heterogenous selection and measurement of outcomes in clinical trials can impair the ability to synthesise results across studies in systematic reviews [4]. Thirdly, in the absence of a set of outcomes that should be measured and reported in all clinical trials in the same condition, it can be difficult to ascertain, in the final publication, whether authors report all results or only those that they find favourable [5],[6].

As a result, the standardisation of outcomes for clinical trials has been proposed as a solution to the problems of inappropriate and non-uniform outcome selection [4],[7] and reporting bias [5],[8]. The most notable work relating to outcome standardisation has been conducted by the OMERACT (Outcome Measures in Rheumatology) collaboration, which advocates the use of core outcome sets designed using consensus techniques that are then measured and reported in clinical trials in rheumatology [9]. However, such initiatives are uncommon. In some specialties, such as paediatrics, the number of conditions covered is low and the quality of existing studies variable [10]. In addition, there is limited guidance in the literature regarding the development of a core outcome set. This paper aims to contribute to the methodology of determining which outcomes to measure in clinical trials, or systematic reviews of clinical trials.

## The Delphi Technique as a Method of Developing Core Outcome Sets

One method for reaching consensus around which outcomes to measure is the Delphi technique, which comprises sequential questionnaires answered anonymously by a panel of participants with relevant expertise. After each questionnaire, the group response is fed back to participants [11]. In terms of the overall validity of the final consensus, this approach has advantages over less structured methods of reaching consensus such as round-table discussions. Participants in a Delphi study do not interact directly with each other, so situations where the group is dominated by the views of certain individuals can be avoided. When participants consider whether to change their opinion or stick to their original answers, after seeing the group response this decision is not affected by the desire to be seen to agree with senior, overly vocal, or domineering individuals. Improvements in global communication have made it feasible to use the Delphi technique to involve geographically distant participants in larger numbers than are traditionally used in studies employing face-to-face discussion, and so it is also increasingly being used to reach consensus around many topics in medicine, such as education, development of clinical guidelines, and prioritisation of research topics.

There is little guidance for researchers who wish to use the Delphi technique, even though aspects of its methodology can be interpreted in a variety of ways. Most published work has provided guidance based on authors' experiences, rather than empirical research or theoretical justification for the methodological decisions made. One systematic review describes a variety of consensus techniques used for designing clinical guidelines [12]. The authors highlighted important methodological decisions that may affect the overall quality of the final consensus, such as the types of participants involved, the questions they are asked, the information they receive to inform their answers, the manner of the interaction between them, and the way in which consensus is agreed. These have also been variously highlighted as important aspects of methodology in other commentaries about the Delphi technique [13]–[15].

To our knowledge, there is no guidance related to methodological considerations or reporting for studies using the Delphi technique to determine which outcomes or domains to measure in clinical research studies. The objective of the systematic review summarised below (and included in full in Text S1) was to examine studies that used the Delphi technique for this purpose. Our recommendations from this review are then summarised to help inform the conduct and reporting of future initiatives.

## A Systematic Review of Studies That Have Used the Delphi Technique to Identify Which Outcomes to Measure in Clinical Trials

We searched Medline (no date restrictions) in January 2010 to identify studies that used the Delphi technique to determine which outcomes to measure in clinical trials or systematic reviews of clinical trials. From each eligible study, the following methodological aspects were noted: the participants involved, the types of questions asked, whether the study was completely anonymised, whether non-responders in earlier rounds were included or excluded from subsequent rounds, and the definition of consensus used by the authors. We also evaluated the quality with which the methods and results were reported. These assessments enabled us to identify variations in the methods applied within these studies, and areas of reporting quality that could be improved.

Of 656 abstracts, 20 full text articles were retrieved, of which five were excluded because they aimed to identify outcomes for use in clinical practice, and the authors did not state whether the participants considered their use in clinical research studies. Many of the 636 studies excluded on the basis of the abstract described the use of the Delphi process to develop clinical guidelines and educational curricula. Of 15 studies included in the review, eight developed core outcome sets for rheumatological conditions. Others identified outcomes for pain in children, degenerative ataxia, gastro oesophageal reflux disease, infantile spasms, maternity care, multiple sclerosis, and thyroid eye disease.

Studies varied in terms of group composition and the manner in which the Delphi process was conducted. Participation in such studies was dominated by researchers, with patients and families seldom involved.

The reporting quality of studies also varied. Important methodological aspects that were generally less well reported were the information provided to participants at the start of the Delphi process, the information fed back to participants after each round, and the level of anonymity. A summary of the reporting quality of the studies is shown in Table 1. Each of the items included in the table had been highlighted, by one or more of the commentaries mentioned earlier [13]–[15], as an important methodological consideration when using the Delphi technique. We tailored the statements so they were relevant for the Delphi process as a method of developing consensus around a core outcome set.

**Table 1:** Reporting quality of the 15 included studies.

Broad Aspect of Reporting	Specific Items for Which the Reporting Quality Was Assessed	Studies in Which Clearly Reported	Studies in Which Not Clearly Reported	N/A
**Size and composition of the panel**	Number of participants	15	0	0
	Types of participants (e.g., clinicians, patients)	15	0	0
	Proportion of each type of participant	15	0	0
	How participants were identified/sampled	14	1	0
**Methodology of the Delphi process**	Administration of questionnaires (e.g., postal)	15	0	0
	How items were generated for first questionnaire	14	1	0
	What was asked in each round	15	0	0
	Information provided to participants before the first round	6	9	0
	How the overall group response was fed back to participants	8	7	0
	Level of anonymity (total or quasi-anonymity)	4	11	0
	A priori definition of “consensus” about whether an outcome should be measured)	7	1	7^a^
	Were non-responders invited to subsequent rounds	10	0	5^b^
**Results**	Number of respondents to each round	14	1	0
	Number who completed every round	11	4	0
	Results for each outcome in each round	0	15	0
	Group response for each outcome (final round)	8	7	0
	Distribution of response for each outcome in the final round	7	8	0
	List of all outcomes that participants agreed should be measured	8	0	7^a^

a Reaching a final consensus was not the aim of the Delphi process, so a definition of consensus was not given.

b All participants responded to each round, so no discussion was made regarding non-responders.

Although an assessment of response rate to each round could be made in 14/15 studies, it was only possible to accurately assess attrition rates in 11/15 studies, which reported the proportion of first round respondents who also completed the final round. Of these, only six studies reported the proportion of participants who completed every round in the Delphi process, from start to finish. Only seven reports presented a measure and distribution of the group opinion for each outcome listed in the final round. No study reported the results, in each round, for every outcome that was considered by the group.

## Guidance about Using the Delphi Technique to Determine Core Outcome Sets

### Involve Clinicians and Patients

Informed clinical decisions can only be based on the results of trials that have measured outcomes of importance to both clinicians and patients. Initiatives to identify which outcomes to measure in clinical trials, however, focus on the opinions of researchers. This means that outcomes included in existing core sets may be selected to serve the needs of researchers in academia or industry, rather than considering how important they are to patients.

Patients have a variety of perspectives about living with a condition, which may differ from those of clinicians and researchers. In one study, involvement of patients in the design of a systematic review highlighted certain outcomes as being of particular importance, but these had not been measured in any of the included trials [16]. Research conducted within the OMERACT group also suggests that clinicians and researchers may not realise that certain outcomes are very important for patients [17]. The perspective of patients is now routinely incorporated into the work conducted by OMERACT [18]. Another important initiative, which actively promotes the involvement of patients and families in identifying priorities in clinical research, is the James Lind Alliance (http://www.lindalliance.org/). In a recent systematic review, this group found a few examples of conditions in which patients and clinicians have worked, together, to identify important research questions [19], and we feel that similar collaboration is necessary to develop core outcome sets. Determining which outcomes are important may be useful to groups who aim to identify important research questions.

The opinions of different groups can be analysed either together or separately. The use of multiple panels, each comprising a different group [17], acknowledges that there may be differences in opinion. If different groups with potentially conflicting views are included in a single panel, they may not be equally represented in the final consensus. This can happen either because the panel includes more participants from a certain group, so the final consensus is numerically dominated by their responses [20], or because participants tailor their answers to agree with a group they perceive to be more authoritative.

In studies that use a single panel, comprising a mixture of participants, authors should report a measure of the distribution of scores for each outcome considered in the final round. This is because cut-off scores, used in most studies, do not describe how strongly the minority feel, and so an apparent consensus could actually be masking major disagreement within the group [13].

### Begin by Asking Open Questions

So that researchers do not impose their views on participants and thus introduce bias into the study, participants are traditionally asked open questions in the first round of a Delphi process. In the context of identifying which outcomes to measure in clinical research studies, this means that participants should suggest potential outcomes that they feel should be considered in the Delphi process, without being prompted or guided by facilitators, steering committees, or reviews of the literature. Most studies we identified did not take this approach. It is not clear whether providing a list to participants for initial consideration may overstate the importance of outcomes that are favourable to the researchers, rather than those which may be of more importance to clinicians and patients. Outcomes measured in previous clinical trials do not always reflect those deemed most appropriate by all stakeholders [1],[2],[21].

### Try to Minimise Attrition

People with minority opinions may be more likely to drop out of studies that use the Delphi process, so attrition as rounds progress can lead to overestimation of the degree of consensus in the final results. Strategies to prevent attrition bias are to only invite people who respond to a pre-Delphi invitation to participate in the first round [22] or to list, in the publication, only those participants who either completed the entire Delphi process, or agreed the final consensus statement [23]. An example of a paragraph that could be used to explain to participants the importance of completing the whole Delphi process is shown in Box 1.

**Box 1. Example Text to Emphasize to Participants the Importance of Completing the Whole Delphi Process**

Thank you for agreeing to participate in our study. It is very important that you complete the questionnaires in each round. The reliability of the results could be compromised if people drop out of the study before it is completed, because they feel that the rest of the group does not share their opinions. If people drop out because they feel their opinions are in the minority, the final results will overestimate how much the sample of participants agreed on this topic.

### Report Certain Aspects of the Methodology and Results

In order to enable appraisal of the quality of studies that use the Delphi process to identify outcomes that should be measured in clinical research, which may in turn affect whether the recommendations are implemented, authors should describe certain important methodological features in the study report. Criticisms of the Delphi technique are that “expertise” of the panel is arbitrarily defined, and that the validity of the final consensus is questionable because individual participants are not accountable for their responses, and they may be led towards conformity with the group, rather than consensus of true opinions [24]. As described earlier, attrition of participants may mean the degree of consensus reached in the final round is overestimated [25]. A recommended checklist of study characteristics and results that should be reported in all studies that use the Delphi technique to determine which outcomes to measure in clinical research studies is shown in Table 2. Given the variation across previous studies, it would be helpful if authors explained their methodological choices, and discussed the effects these may have on the results.

**Table 2:** Recommended checklist that should be reported in studies using the Delphi technique to determine which outcomes to measure in clinical trials or systematic reviews.

**Size and Composition of the Panel**
The total number of participants invited, and the number who completed the first round
Whether the following types of participants were involved in the study: clinicians (and whether they were eligible on the basis of treating patients with the condition of interest, or whether clinical trial involvement was an additional requirement), patients or their families, researchers, biostatisticians, representatives from the pharmaceutical industry, representatives from drug regulatory authorities, or other types of participants.
The proportion of each type of participant described above
How participants were identified/sampled
**Methodology of the Delphi Process**
Administration of questionnaires: postal, email, Internet, in person (e.g., at a clinic), or at a meeting
Information about outcomes, known to the facilitators before the study, which was provided to participants before the first round: e.g., if the Delphi process followed a review of outcomes measured in clinical trials, were the results of the review shared with participants? Alternatively, if some work had been conducted prior to the Delphi (e.g., workshop meeting, or focus groups amongst patients), were the results presented to the participants?
How outcomes were considered in the first questionnaire: were participants asked an open question i.e., no outcomes were initially listed, or were they asked to comment on a pre-specified list? If the latter, was the source of the list identified? Where possible, the questions asked to participants should be described in the methods, or made available to the reader, as supplementary information.
What was asked in subsequent rounds: where possible, the questions asked to participants should be described in the methods, or made available to the reader, as supplementary information
Feedback to participants after each round: if the results were not fed back, but only certain outcomes were carried forward to the next round (e.g., only those suggested by at least 10% were carried forward), this should be clearly described
Level of anonymity should be described: In order to be “fully anonymised”, participants should not know the identities of the other individuals in the group, nor should they know the specific answers that any other individual gave. In studies that are “quasi-anonymised”, the participants know the identities of some or all of the other individuals, but do not know how they individually responded to any of the questions in any round. In studies that are not anonymised, participants know the identity of some or all of the other individuals, and also know how some or all of them responded to any of the questions in any round.
If a pre-determined definition of consensus was used, this should be clearly described in the methods section of the study report
Were non-responders invited to subsequent rounds, or were they excluded from the rest of the study? Were additional people invited as the Delphi progressed?
**Results**
Number of participants invited to each round
Number who completed every round
Results for each outcome scored by participants in each round: a measure of group response, preferably with a measure of distribution. If these data cannot be included in the publication, even as a supplementary file, it should be made available on request.
Measure of group response for each outcome scored by participants in the final round
Distribution of response for each outcome scored by participants in the final round
A comprehensive list of all the outcomes that participants agreed should be included in the core set

## Determining How to Measure the Outcomes Included in the Core Set

Following the determination of which outcomes to include in a core set, guidance is then required as to how to measure them. One established method for doing so is the OMERACT approach. Once core outcomes are agreed upon, potential instruments to measure them are identified. The psychometric properties of these instruments are then reviewed in terms of feasibility, validity, and responsiveness before the preferred instruments are agreed [9]. A more detailed review of the possible approaches to this question of how to measure the chosen outcomes is beyond the scope of this paper.

## Future Areas of Methodological Research

Given variations in methodology between studies, we feel there is a need for research to determine how best to develop core outcome sets. An agenda for this research could be designed through the COMET Initiative (Core Outcome Measures for Effectiveness Trials), which is an international network of individuals and organisations with interest or experience in the development, application, and promotion of core outcome sets (http://www.liv.ac.uk/nwhtmr/comet/comet.htm). One such area of ongoing research and discussion relates to whether core outcome sets designed for clinical practice, such as those developed in the five studies we excluded, should be the same as those designed for research. Another priority is research to identify the most effective ways to incorporate the views of different groups of participants, especially patients, in the design of core outcome sets.

## Supporting Information

Text S1 Full report (and PRISMA checklist) of the systematic review of studies that used the Delphi technique to determine which outcomes to measure in clinical trials.
(0.87 MB DOC)
