# Supplementary material for: Using the Delphi Technique to Determine Which Outcomes to Measure in Clinical Trials: Recommendations for the Future Based on a Systematic Review of Existing Studies
Source: PLoS Med. 2011 Jan 25;8(1):e1000393. doi: 10.1371/journal.pmed.1000393 (PMC3026691; doi:10.1371/journal.pmed.1000393)
Supplement: Text S1 — Full report (and PRISMA checklist) of the systematic review of studies that used the Delphi technique to determine which outcomes to measure in clinical trials. (0.87 MB DOC) [file pmed.1000393.s001.doc]

**Using the Delphi technique to determine which outcomes to measure in clinical trials: a systematic review of existing studies and recommendations for the future**

**Ian Sinha^1^, Rosalind L. Smyth^1^, Paula R. Williamson^2^**

**1 University of Liverpool, Alder Hey Children's Hospital, Liverpool, L12 2AP, UK**

**2 Centre for Medical Statistics and Health Evaluation, Faculty of Medicine, University of Liverpool, Brownlow Street, Liverpool, L69 3GS**

**Abstract**

**Background**

The development of core outcome sets for use in clinical trials has been advocated, as a way of ensuring appropriate outcomes are measured, reducing heterogeneity between studies, and preventing outcome reporting bias. There is little guidance relating to the use of the Delphi technique for reaching consensus around which outcomes should be measured in clinical trials. Issues which may affect the credibility of the results of studies which use the Delphi technique for this purpose include inappropriate group composition, poor questioning technique, attrition bias, analysis which can miss or overstate the importance of certain outcomes, and incomplete reporting of results.

Our aim was to systematically review studies that use the Delphi technique to determine which outcomes to measure in clinical trials or systematic reviews of clinical trials, to identify variations in the methods applied within these studies.

**Methods and findings**

We searched Medline (no date restrictions) in January 2010, for relevant studies. From these, we extracted data on methodological aspects including the participants involved, the types of questions they were asked, whether the study was completely anonymised, whether non-responders in earlier rounds were included or excluded from subsequent rounds and the definition of consensus used by the authors.

The literature search identified 656 potentially relevant abstracts. Of these, 20 were retrieved, and 15 were included in the review. Eight of these developed core outcome sets for rheumatological conditions, and others identified outcomes for pain in children, degenerative ataxia, gastro oesophageal reflux disease, infantile spasms, maternity care, multiple sclerosis, and thyroid eye disease. There was variation between the studies in terms of the composition of the groups, and the manner in which the Delphi process was conducted. The reporting quality of studies was variable. Patients were rarely involved in the process of determining which outcomes to measure in clinical trials.

**Conclusions**

Researchers who use the Delphi technique to design core outcome sets should be aware of issues which may affect the credibility of their study, and explain methodological decisions, in relation to the study aims, in the main publication. There is need for methodological research to determine the best way to design core outcome sets.

**Introduction**

Good clinical trial design requires researchers to specify in advance, in the protocol, those outcomes to be measured. If research has not been conducted to identify the most appropriate clinical trial outcomes in a given condition, three problems may impair the usefulness of the research in informing clinical practice. Firstly, researchers can select outcomes which suit their needs, at the expense of outcomes which are of most importance to patients or clinicians [1,2,3]. Secondly, heterogenous selection and measurement of outcomes in clinical trials can impair the ability to synthesise results across studies in systematic reviews [4]. Thirdly, in the absence of a set of outcomes that should be measured and reported in all clinical trials in the same condition, it can be difficult to ascertain, in the final publication, whether authors report all results or only those which they find favourable [5,6].

The standardisation of outcomes for clinical trials has, therefore, been proposed as a solution to the problems of inappropriate and non-uniform outcome selection [4,7,8] and reporting bias [5,9,10]. The most notable work relating to outcome standardisation has been conducted by the OMERACT collaboration, which advocates the use of core outcome sets, designed using consensus techniques, which are measured and reported in clinical trials in rheumatology [11]. However, such initiatives are uncommon. In some specialties, such as paediatrics, the number of conditions covered is low and the quality of existing studies variable [12].

One method for reaching consensus around which outcomes to measure is the Delphi technique, which comprises sequential questionnaires, answered anonymously, by a panel of participants, who each have relevant expertise. After each questionnaire, the group response is fed back to participants [13]. With regard to the overall validity of the final consensus, this approach has advantages over less structured methods of reaching consensus, such as round-table discussions. Participants in a Delphi study do not interact directly with each other, so situations where the group is dominated by the views of certain individuals can be avoided. When participants consider whether to change their opinion or stick to their original answers, after seeing the group response, this decision is not affected by the desire to be seen to agree with senior, overly vocal, or domineering individuals. Improvements in global communication have made it feasible to use the Delphi technique to involve geographically distant participants, in larger numbers than are traditionally used in studies employing face-to-face discussion, and so it is also increasingly being used to reach consensus around other topics in medicine, such as education [14], development of clinical guidelines [15] and prioritisation of research topics [16].

There is little guidance for researchers who wish to use the Delphi technique, even though aspects of its methodology can be interpreted in a variety of ways. Most published work has provided guidance which is based on authors’ experiences, rather than empirical research or theoretical justification for the methodological decisions made. One systematic review describes a variety of consensus techniques which are used for designing clinical guidelines [17]. The authors highlighted important methodological decisions which may affect the overall quality of the final consensus. These were: the types of participants involved, the questions they are asked, the information they receive to inform their answers, the manner of the interaction between them, and the way in which consensus is agreed. To our knowledge, there is no guidance related to methodological considerations or reporting for studies using the Delphi technique to determine which outcomes or domains to measure in clinical trials or systematic reviews.

The objectives of this study were:

1) To systematically review studies that use the Delphi technique to determine which outcomes, or outcome domains, to measure in clinical trials or systematic reviews of clinical trials, to identify variations in the methods applied within these studies.

2) To discuss improvements to the design and reporting of future studies, based on the findings of our review, for people who wish to use the Delphi technique for this purpose.

**Methods**

Study selection

Included studies

We included two types of studies that used the Delphi technique to determine which outcomes or outcome domains to measure in clinical trials or systematic reviews of clinical trials. We included studies in which the final consensus was determined using the Delphi process and we included studies that used the Delphi technique to identify the opinion of a group of panelists, but not in order to reach a final consensus about which outcomes to measure. This latter category includes studies in which the results of the Delphi technique were used to inform participants in subsequent meetings to determine consensus, or to determine which outcomes or domains to measure in specific trials or systematic reviews.

We felt it was appropriate to combine studies relating to outcomes with those relating to outcome domains, because we expected the methods used in both these types of studies to be sufficiently similar.

Excluded studies

We excluded studies that did not specifically state that the outcomes selected could be used in clinical research studies. For example, studies identifying outcomes to evaluate the quality of care given by healthcare providers, or to guide the management of individual patients in clinical practice, were not included, unless the authors stated that participants considered their use in clinical research.

Identification of studies

We identified relevant studies by searching Medline in January 2010 (from 1950 to January 2010). The following search strategy was used: “((outcome$ OR endpoint$ OR end point$ OR variable$ OR domain$) AND (Delphi OR Delphi method OR Delphi technique$ OR sequential questionnaire$)).mp.”.

Data extraction

The following methodological aspects were identified from each study report: (1) who was involved in the Delphi process, in terms of the number of clinicians, patients and other participants, and their geographical location; (2) the types of questions participants were asked; (3) whether the study was completely anonymised (participants’ identities and answers were hidden from the group) or quasi-anonymised (identities of participants were disclosed to the group, but individual answers were kept hidden); (4) whether non-responders in earlier rounds were included or excluded from subsequent rounds; (5) the definition of consensus used by the authors.

We contacted the corresponding author by email to obtain information which was not available or clear in the study report.

Assessment of reporting quality

We assessed in the study report whether the following methodological aspects were described: the number of participants invited to each round; the types of participants involved, and the proportion of each type; how these participants were initially identified as being eligible for the study; the method by which the Delphi study was conducted (eg postal, email, internet); what information participants received before the first round; how outcomes included in the first round of questions were identified (ie were they identified before the study, or did participants suggest outcomes that should be considered by the group); whether the study was completely anonymised or quasi-anonymised; what was asked in each round; what feedback was provided to participants after each round; and how the authors determined, for each outcome, whether consensus had been reached as to whether it should be measured. If any of these were not described to the level of detail that would permit another researcher to reproduce the methodology, we classed the reporting of the methodological aspect as unclear. We also identified whether the authors explained these methodological choices.

With regard to the reporting of results, we assessed whether the authors stated the number of respondents to each round, the proportion of participants who completed every round in the whole Delphi process from start to finish, the results for each outcome in each round, a measure of group response and distribution for each outcome in the final round, and a list of all outcomes that the group decided should be measured in clinical research studies.

Data analysis and presentation of results

For synthesis of data we described the studies narratively and tabulated their characteristics. Consistent with the nature of the data, the results are presented in textual format.

**Results**

Identification of studies

The review flowchart is shown in Figure 1. Of twenty studies for which the full text study report was retrieved, five were excluded because they aimed to identify outcomes for use in clinical practice, and the authors did not state whether the participants considered their use in clinical research studies [18,19,20,21,22].

Fifteen studies were included in the review [7,23,24,25,26,27,28,29,30,31,32,33,34,35,36]. Eight of these developed core outcome sets for rheumatological conditions, of which five were conducted by the OMERACT group (relating to gout [35], systemic sclerosis [28] and associated pulmonary hypertension [25], fibromyalgia [31]and psoriatic arthropathy [34]), and three were conducted by other collaborations (relating to idiopathic inflammatory myopathy [7], juvenile systemic lupus erythematosis [32], and ankylosing spondylitis[36]). Others developed core outcome sets for pain in children [30], degenerative ataxia [33], gastro oesophageal reflux disease [23], infantile spasms [29], maternity care [24], multiple sclerosis [27], and thyroid eye disease [26]. The methods and results of one study were described across two publications, of which the one that describe the study most comprehensively [7] is included in this review, and the other is referenced as an additional article [37]. One study was conducted in two distinct stages, both of which were reported in separate publications. The most recent publication describes both stages of the study [31], and is included in this review, while the earlier publication is referenced as an additional article [38].

Eight studies used the Delphi technique as the main method of reaching consensus about which outcomes to measure in clinical research studies. Four of these related to outcomes in rheumatological conditions (of which three were conducted by the OMERACT collaboration [25,28,35] and one by another group [36]), two related to neurological conditions [27,29], one related to gastroenterology [23] and one to maternity care [24]. The remaining seven studies used the results of a Delphi process either to inform people involved in subsequent consensus studies [7,26,30,31,32,34], or to help design a specific systematic review [33].

Reporting quality

The reporting quality of the studies is summarised in Table 1.

Reporting of methods

The types of participants involved, and the size and composition of the group, were adequately described in all studies. All but one study described the way in which, at the start of the Delphi process, an initial list of outcomes was identified, for consideration by the panel. All studies described the questions which were asked, during the rounds of the Delphi process, to determine which of these should be measured in clinical trials. All studies explained, when applicable, the pre-determined definition of ‘consensus’ about whether an outcome should be measured in clinical trials. Important methodological aspects which were generally less well reported were the information provided to participants at the start of the Delphi process (clearly reported in 6/15 studies), the information which was fed back to participants after each round (clearly reported in 8/15 studies), and the level of anonymity in the study (clearly reported in 4/15 studies).

Reporting of results

An assessment of response rate could be made in 14/15 studies, which reported, for each round, the number of invited participants who responded. It was possible to make an assessment of attrition rates in 11/15 studies, which reported the proportion of first round respondents who also completed the final round. Of these, six studies reported the proportion of participants who completed every round in the Delphi process, from start to finish.

Although all studies provided a list of outcomes that participants in the Delphi process felt should be measured in clinical research studies, only eight reports presented a measure of the group opinion for each outcome listed in the final round, and seven of these also reported the distribution of scores for each item. No study reported the results, in each round, for every outcome that was considered by the group.

Explanation for methods adopted in the study report, or discussion of the impact of methodological decisions on the final results

The composition of the groups was discussed, in ten studies, but only six discussed the implications of the size of their group on the external validity of their results. Two studies discussed why they chose to either identify outcomes in a certain way or to not identify any outcomes at the start of the Delphi process. Five studies explained the methods used to determine which of these initial outcomes should be measured in clinical trials. Five studies discussed why they defined consensus in a certain way. Two studies discussed the implications of presenting results to participants in a certain way.

Methodological variation between the studies

Composition of the group

Table 2 shows the size and composition of the groups. The group size varied from 13 [23] to 222 [32]. Generally, studies conducted through clinical or research networks involved more participants.

Clinicians were included in all but one study, which only involved patients [33]. Eight studies were conducted through clinical trial networks [7,28,29,31,32,34,35,36], four only involved clinicians who had published research in the relevant field [23,25,29,30], and three involved both clinicians and researchers [24,26,27].

Four groups involved patients or families [24,33,36,38]. Some groups involved other types of participants, including health service managers [24], pharmaceutical industry employees [30,35]), and drug regulatory agency representatives (The US Food and Drug Administration) [30].

Five studies involved different types of participants, of which four used a single panel comprising a mix of the groups [24,30,35,36]. In the other study, relating to a core set of outcomes for fibromyalgia, clinicians and patients completed two separate Delphi studies, which were used to inform discussions at a subsequent consensus meeting [31,38].

Anonymity

The level of anonymity is summarised in Table 3. In thirteen studies conducted by email, post or internet, seven were conducted completely anonymously (participants were not aware who the other members of the group were, and individuals’ answers were kept secret from the group) [24,25,29,30,32,33,35], and in the others complete anonymity is presumed (it is unclear whether participants knew the identities of other individuals). In the two studies in which the Delphi process was conducted at face-to-face meetings, voting was anonymous in one [23] but not the other [27].

In nine studies, participants met before [7,35], during [23,27], after [26,30,32,36], or before and after [31] the Delphi process.

Structure of the Delphi process

The general format involved identification of potential outcomes, followed by determination of those which were most important or appropriate for clinical trials.

**Identification of potential outcomes**

In four studies, outcomes were suggested by participants, without prompting or guidance from the facilitators [26,30,32,33]. In two studies, participants suggested outcomes within a framework of domains, suggested by a steering group [28], or based on international recommendations [27].

In four studies, outcomes were proposed by a steering committee [25], or by facilitators who , by reviewing the literature, identified outcomes used in previous studies [29,35,36]. In three studies, outcomes measured in clinical trials were discussed at international meetings, and a list of potentially eligible outcomes for the Delphi surveys were identified [7,23,35]. In one study, outcomes were identified from both a systematic review of clinical trials and by asking participants to suggest two ‘new’ outcomes that were not listed [24] In another, the outcomes considered by clinicians were suggested by a steering group, and those considered by patients were identified in focus groups (which were conducted amongst patients, prior to the survey) . [31].

**Determining the importance of potential outcomes**

Participants either scored the importance of each outcome [24,25,26,28,33,35], voted for or against its measurement in clinical trials [7,23,27,29,36], distributed a set number of points amongst outcomes, according to importance [31,34], or ranked outcomes in order of importance [30,32]. In two studies [27,29] participants were asked to justify their answers.

**Feedback of the results to participants**

Eight groups fed back either the average score for each outcome [24,25,26,31,33,34,35], or the percentage of people voting for its inclusion in the core set [27]. In four studies, facilitators analysed data and presented a new list of outcomes, without presenting a measure of group opinion to participants [28,29] [30,32]. In three studies it was unclear what measure of group response was fed back to participants [7,23,36].

How consensus was reached about which outcomes to measure

The decisions about which of the outcomes that were initially suggested should be measured in clinical research studies are summarised in Table 4. Eight studies used the Delphi technique as the main method of reaching consensus about which outcomes to measure, of which six recommended outcomes which received a pre-determined score [28,35], or a pre-determined proportion of participants felt it should be included in a core set [23,27,29,36]. These scores and proportions varied between the six studies. In another study [24], an outcome was included in the core set if its score was higher than the mean score of all outcomes and at least 70% of participants scored it 4/5 on a Likert-type scale. In one study, the final core set of outcomes was determined by a steering group [25].

Seven studies did not use the Delphi process itself to reach consensus about which outcomes to measure. Six of these used the results of the Delphi to inform people participating in subsequent consensus studies which aimed to design core outcome sets. Two of these studies [26,32] used the Delphi process as a way of filtering out outcomes felt to be less important, and these were not considered at the subsequent consensus meeting. In the other four studies [7,30,31,34], each outcome considered in the Delphi process was carried forward to the subsequent consensus meeting, regardless of its score. The aim of one study was to inform the design of a systematic review [33], and all the outcomes which were considered are ranked in order of importance, but the final decision about whether they should be measured in the review is not described.

Attrition of participants

In four studies, no participants who completed the first round subsequently dropped out of the study [23,27,30,33], and in one study the only participants who did so completed all but the final round of the study [34]. In the remaining ten studies, some participants dropped out of the Delphi process before the final round. In five of these, [25,29,31,32,36] each participant was invited to every round, even if they did not complete previous questionnaires. In three studies [24,28,35] people who did not respond to any given round were excluded from the remainder of the study. In two studies, additional participants were invited as the Delphi progressed [7,26].

**Discussion**

Across studies using the Delphi technique to identify which outcomes to measure in clinical research studies, we found marked variation in the methods used, and the quality of reporting of key aspects. Participation in such studies is dominated by researchers, with patients and families seldom involved.

Composition of the groups

Informed clinical decisions can only be based on the results of trials which have measured outcomes of importance to both clinicians and patients. Initiatives to identify which outcomes to measure in clinical trials, however, focus on the opinions of researchers. This means that outcomes included in existing core sets may be selected to serve the needs of researchers in academia or industry, rather than considering how important they are to patients.

Patients, who have a unique perspective about living with a condition, should be asked which outcomes they feel are of most importance. Outcomes important to clinicians or researchers may differ from those used by patients. In one study, involvement of patients in the design of a systematic review highlighted certain outcomes as being of particular importance, but these had not been measured in any of the included trials [33]. Research conducted within the OMERACT group also suggests that clinicians and researchers may not realise that certain outcomes are very important for patients [31,39]. The perspective of patients is now routinely incorporated into the work conducted by OMERACT [40].

The opinions of different groups can be analysed either together or separately. The use of multiple panels, each comprising a different group [31,38], acknowledges that there may be differences in opinion. If different groups with potentially conflicting views are included in a single panel, they may not be equally represented in the final consensus. This can happen either because the panel includes more participants from a certain group, so the final consensus is numerically dominated by their responses [24], or because participants tailor their answers to agree with a group which they percieve to be more authoritative [33].

In studies which use a single panel, comprising a mixture of participants, authors should report a measure of the distribution of scores for each outcome considered in the final round. This is because cut-off scores, used in most studies, do not describe how strongly the minority feel, and so an apparent consensus could actually be masking major disagreement within the group [41].

Avoiding bias introduced by researchers and facilitators

So that researchers do not impose their views on participants, and thus introduce bias into the study, participants are traditionally asked open questions in the first round of a Delphi process. In the context of identifying which outcomes to measure in clinical research studies, this would translate into participants suggesting potential outcomes that they feel should be considered in the Delphi process, and would not be prompted or guided by facilitators, steering committees or reviews of the literature. Most studies we identified did not take this approach. It is not clear whether providing a list to participants for initial consideration may overstate the importance of outcomes which are favourable to the researchers, at the expense of others, which may be of more importance to clinicians and patients. It is known that outcomes measured in previous clinical trials do not always reflect those deemed most appropriate by all stakeholders [1,2,42].

Avoiding bias occurring because of participant attrition

People with minority opinions may be more likely to drop out of studies which use the Delphi process, so attrition as rounds progress can lead to overestimation of the degree of consensus in the final results. Strategies to prevent attrition bias are to only invite people who respond to a pre-Delphi invitation to participate in the first round [32] or to list, in the final publication, only those participants who either completed the entire Delphi process or agreed the final consensus statement [29].

Robustness of this review

Half of the studies we identified related to rheumatological conditions. Similarities in the methods used in these studies may have skewed our findings towards those proposed by the OMERACT group. However we did identify differences between the studies, in terms of methodological decisions and reporting quality.

Suggested aspects of the methodology and results that should be reported

In order to enable appraisal of the quality of studies which use the Delphi process to identify outcomes that should be measured in clinical research, which may in turn affect whether the recommendations are implemented, authors should describe certain important methodological features in the study report. Criticisms of the Delphi technique are that ‘expertise’ of the panel is arbitrarily defined, and the validity of the final consensus is questionable because individual participants are not accountable for their responses, and they may be led towards conformity with the group, rather than consensus of true opinions [43]. It has also been suggested that people who drop out of studies that use the Delphi technique are more likely to hold opinions which differ from the majority view, and so the degree of consensus reached in the final round may be overestimated [44].

To allow the reader to assess whether the composition of the panel was appropriate, authors should report the number and types of participants involved in the study, and how they were identified. To enable assessment of the Delphi process itself, they should report how potential outcomes were identified at the start of the study, and the process of determining which of these should be measured in clinical trials. Such details must include the types of questions asked, and how authors decided whether consensus had been reached amongst the group members.

To enable assessment of the risk of bias from attrition of participants, authors should report whether or not non-responders were invited to continue in the study or whether they were excluded. They should also report the number of people who dropped out in each round.

As a minimum, the results should describe the group opinion for each outcome which was included in the final round. They should describe a measure of the group response (eg average score) and distribution (eg interquartile range).

Finally, given the variations between studies, it would be helpful if authors explained the rationale behind their methodological choices, or discussed the effects these may have on the results.

Future areas of methodological research

Given variations in methodology between studies, we feel there is a need for research to determine how best to develop core outcome sets. An agenda for this research could be designed through the COMET initiative (Core Outcome Measures for Effectiveness Trials), which is an international network of individuals and organisations with interest or experience of the development, application and promotion of core outcome sets (<http://www.liv.ac.uk/nwhtmr/comet/comet.htm>). One such area of ongoing research and discussion relates to whether core outcome sets designed for clinical practice, such as those developed in the five studies we excluded [18,19,20,21,22], should be the same as those designed for research. Another priority is research to identify the most effective ways to incorporate the views of different groups of participants, especially patients, in the design of core outcome sets.

**Summary**

Studies which use the Delphi process for the purpose of gaining consensus around a core outcome set for clinical trials are conducted to improve the quality and usefulness of clinical research, and in order for their recommendations to be implemented, they should be of sufficiently high quality. Where possible patients and clinicians should be involved. Researchers facilitating these studies should avoid imposing their views on participants.

Poor reporting of methodological decisions makes it difficult to appraise these studies. Researchers wishing to use the Delphi technique for this purpose should be aware of these issues when designing their study, and explain methodological decisions, in relation to the study aims, in the main publication.

Figures

Figure 1 - flowchart of the review


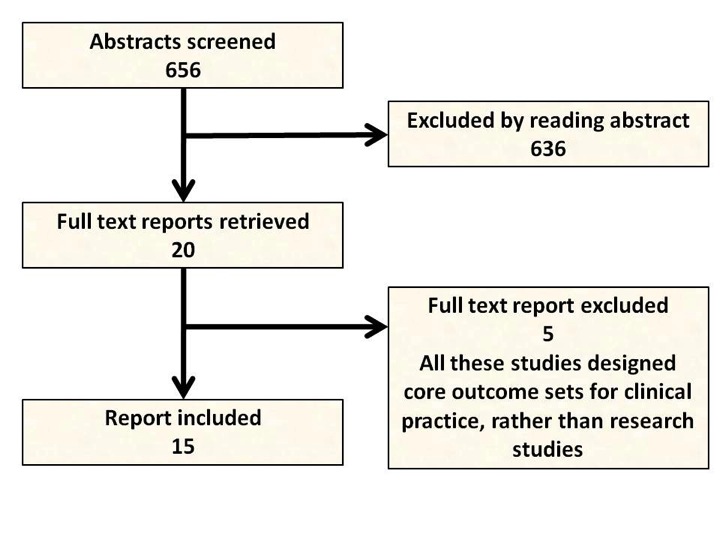


Tables

Table 1 - reporting quality of the 15 included studies

|  | **Studies in which clearly reported** | **Studies in which not clearly reported** | **N/A** |
| --- | --- | --- | --- |
| **Size and composition of the panel** |  |  |  |
| Number of participants | 15 | 0 | 0 |
| Types of participants (eg clinicians, patients) | 15 | 0 | 0 |
| Proportion of each type of participant | 15 | 0 | 0 |
| How participants were identified/sampled | 14 | 1 | 0 |
| **Methodology of the Delphi process** |  |  |  |
| Administration of questionnaires (eg postal) | 15 | 0 | 0 |
| How items were generated for first questionnaire | 14 | 1 | 0 |
| What was asked in each round | 15 | 0 | 0 |
| Information provided to participants before the first round | 6 | 9 | 0 |
| How the overall group response was fed back to participants | 8 | 7 | 0 |
| Level of anonymity (total or quasi-anonymity) | 4 | 11 | 0 |
| A priori definition of ‘consensus’ about whether an outcome should be measured) | 7 | 1 | 7 _a_ |
| Were non-responders invited to subsequent rounds | 10 | 0 | 5 _b_ |
| **Results** |  |  |  |
| Number of respondents to each round | 14 | 1 | 0 |
| Number who completed every round | 11 | 4 | 0 |
| Results for each outcome in each round | 0 | 15 | 0 |
| Group response for each outcome (final round) | 8 | 7 | 0 |
| Distribution of response for each outcome in the final round | 7 | 8 | 0 |
| List of all outcomes that participants agreed should be measured | 8 | 0 | 7_a_ |

Footnotes to Table 1:

a: Reaching a final consensus was not the aim of the Delphi process, so a definition of consensus was not given

b: All participants responded to each round so no discussion was made regarding non-responders

Table 2 - composition of the groups involved in the Delphi processes

| **Study** | **Method of identification of the sample of participants** | **Number responding to Round 1** | **Number (%) of participants who were health care providers** | **Number (%) of all participants who were patients** | **Other types of participants involved in the study** | **Research experience of the panel** | **Number of countries represented (Continents)** |
| --- | --- | --- | --- | --- | --- | --- | --- |
| **Dent [23]** | People known to facilitator | 13 | 12 (92) | 0 | Clinical trial methodologist n=1 (8) | 1 member (non-clinician) was ‘an expert in RCT methods’; | 9 (North America, Europe, Asia, Australasia) |
| **Devane [24]** | Health professional network; patient groups | 218 | 147 (68) | 24 (11) | Health service managers n=14(6); epidemiologists n=9(4); ‘other’ _a_ 24 (11) | 78/218 (36%) self- identified as researcher | 13 (North America, South America, Europe, Asia, Australasia, Africa) |
| **Distler [25]** | Clinical trial network | 69 | 69 (100) | 0 | 0 | All had published clinical research | Unclear (North America, Europe, Australasia, Asia) |
| **Douglas [26]** | Health professional network | 84 | 84 (100) | 0 |  | Unclear | 14 (North America, Europe, Australasia, Asia) |
| **Khan [27]** | Local professionals | 23 | 23 (100) | 0 | 0 | Unclear | 1 (Australia) |
| **Khanna [28]** | Clinical trial network | 62 | 62 (100) | 0 | 0 | All were members of a clinical trial network. | Unclear (North America, South America, Europe, Asia) |
| **Lux [29]** | Published researchers | 31 | 31 (100) | 0 | 0 | All participants had presented/ published clinical research | 15 (North America, South America, Europe, Asia) |
| **Mease 2008 [31]** | Clinical trial network; Local patients | 96 | 23 (24) | 73 (76) | 0 | All clinicians were members of a clinical trial network. | 1 (North America) Nb consensus meeting at OMERACT, after the clinician Delphi, was multinational |
| **McGrath [30]** | People known to facilitator | 26 | 17 (65) | 0 | FDA/NIH n=5 (19); industry n=4 (16) | All doctors were clinical researchers. | 4 (North America, Europe) |
| **Miller [7]** | Clinical trial network | 70 | 70 (100) | 0 | 0 | All were investigators in a clinical trial network | 14 (North America, south America, Europe, Asia) |
| **cont** | **Identification of participants** | **Number in Round 1** | **Number(%) of healthcare providers** | **Number (%) of patients** | **Other participants** | **Research experience of the panel** | **Number of countries (Continents)** |
| **Ruperto [32]** | Clinical trial network | 222 | 222 (100) | 0 | 0 | All were members of clinical trial networks. | 46 (North America, South America, Europe, Asia, Australasia, Africa) |
| **Serrano 2009 [33]** | Identified by patient groups | 53 | 0 | 53 (100) | 0 | All participants were patients | 1 (Europe) |
| **Taylor 2005 [34]** | Clinical trial network | 32 | 32 (100) | 0 | 0 | Most were members of a clinical trial network | 10 (North America, Europe, Australasia, Africa) |
| **Taylor 2008 [35]** | Clinical trial network | 33 | 30 (91) | 0 | Industry n=3 (9) | Most participants were members of OMERACT | 11 (North America, South America, Europe, Asia, Australasia) |
| **Zochling [36]** | Clinical trial network | 55 | 53 (96) | 2 (4) | 0 | All were members of a clinical trial network | Unclear (unclear) |

Footnotes to Table 2:

a: anaesthetists, social scientists and lactation specialists (numbers of each group unknown)

b: 23 clinicians completed all 3 rounds. Unclear how many completed first round

c: 322 responded to at least one round of 12 different Delphi studies. Unclear how many people participated in more than one study, so the total number of people involved is unclear

Table 3 - Level of anonymity in the Delphi studies

| **Study** | **Number of rounds** | **How Delphi was conducted** | **Did participants meet** | **Did participants know the identity of other group members?** | **Did participants know the answers provided by other individuals in the group?** |  |
| --- | --- | --- | --- | --- | --- | --- |
| **Dent [23]** | 3 | Meeting | Yes _a_ | Yes | No | Quasi |
| **Devane [24]** | 3 | Internet | No | Unclear | Unclear | Complete |
| **Distler [25]** | 3 | Internet | No _b_ | No | No | Complete |
| **Douglas [26]** | 3 | Email | Yes _c_ | Unclear | No | Complete |
| **Khan [27]** | 3 | Meeting | Yes _a_ | Yes | Yes | Not |
| **Khanna [28]** | 3 | Email | No _b_ | Unclear | Unclear | Unclear |
| **Lux [29]** | 6 | Email | No | No | No | Complete |
| **Mease 2008 [31]** |  | Unclear | Yes  _d_ | Unclear | No | Presumed |
| **McGrath [30]** | 2 | Email | Yes _c_ | No | No | Complete |
| **Miller [7]** | 2 | Email | Yes  _e_ | Unclear | No | Presumed |
| **Ruperto [32]** | 2 | Postal | Yes _c_ | Unclear | Unclear | Complete |
| **Serrano [33]** | 3 | Email | No | No | No | Complete |
| **Taylor 2005 [34]** | 3 | Email | No | Unclear | Unclear | Unclear |
| **Taylor 2008 [35]** | 3 | Internet | Yes  _e_ | No | No | Complete |
| **Zochling [36]** | 3 | Email | Yes _f_ | Unclear | Unclear | Unclear |

Footnotes to Table 3:

a: at least part of the Delphi process was conducted at a meeting

b: steering group met but did not participate in the Delphi process

c: Results of Delphi process were used to inform participants at a subsequent consensus meeting

d: Delphi was preceded by focus groups which did not constitute part of the Delphi process itself

e: Delphi was preceded by meetings which did not constitute part of the Delphi process itself

f: The results of the Delphi process were presented at a conference, involving participants, and the list of core outcomes was refined

Table 4 – How it was ultimately determined which outcomes should be measured.

| **Study** | **Was Delphi the final method for reaching consensus, or was it followed by another consensus process** | **Definition of consensus** | **Initial number of outcomes/ domains** | **Number of outcomes/ domains recommended** |
| --- | --- | --- | --- | --- |
| **Dent [23]** | Delphi was used to write consensus document about trial design (including statements about outcomes) | Consensus was reached if at least 75% of participants agreed with a statement that an outcome should be measured | n/a _a_ | n/a _a_ |
| **Devane [24]** | Delphi was final method for reaching consensus | After each round, the only outcomes included for further consideration were those whose mean score amongst participants was higher than the mean score of all outcomes, and at least 70% of participants scored it 4/5 on a Likert-type scale. After the final round, the outcomes which met this criteria were included in the core set | 263 outcomes | 48 outcomes |
| **Distler [25]** | Delphi was final method for reaching consensus | The ultimate core set was selected by a steering group, after the final round of the Delphi. This was done using cluster analysis, in which outcome domains suggested by the group as being most important were classed as feasible or not | 17 domains | 7 domains |
| **Douglas [26]** | Delphi process was followed by a meeting at which NGT was used to determine the final core set | After the first and second rounds, outcomes with a median score of 3/9 or less on a Likert-type scale were removed. After the final round, only outcomes with a median score of at least 6/9 on a Likert-type scale were considered in a subsequent consensus meeting. At this meeting, 80% of participants had to agree for an outcome to be included in the core set. | 220 outcomes | 40 outcomes |
| **Khan [27]** | Delphi was final method for reaching consensus | After the first round, outcomes voted for by less than 50% of participants were removed. After the final round, outcomes voted for by at least 50% of participants were included in the core set | 144 outcomes | 30 outcomes |
| **Khanna [28]** | Delphi was final method for reaching consensus | After the first two rounds, a nine-person steering committee filtered out outcomes they felt were not appropriate. After the second round of the Delphi process, this steering committee considered each outcome, and chose whether it should still be considered. Outcomes rejected by at least 33% of participants were removed. In Round 3, all participants considered each outcome and scored, on a scale of 1-9, whether it should be included in the core set (9=should be included). Outcomes with a median score of at least 7 were included in the core set | 212 outcomes | 31 outcomes |
| **Lux [29]** | Delphi was used to write consensus document about trial design (including statements about outcomes) | There was no pre-defined level of consensus about statements relating to which outcomes should be measured. Instead, the participants agreed on the overall recommendations at the end of the Delphi process. | n/a _a_ | n/a _a_ |
| **Mease 2008 [31]** | Results of Delphi process were used to help participants at subsequent meetings decide which outcome domains to measure | There were no pre-defined cut-off scores for domains to be considered, at the consensus meeting involving clincians and researchers, for inclusion in the core set. The participants at that meeting suggested that outcome domains which at least 50% of participants felt to be important should be considered ‘key’ domains for clinical trials. The top 15 domains considered in the separate patient Delphi process were listed | 40 domains presented to clinicians. 104 domains presented to patients | 8 ‘key’ domains and 5 ‘important’ domains from clinicians. ‘Top 15’ domains from patients |
| **McGrath [30]** | Results of Delphi process were used to help participants at subsequent meetings decide which outcomes to measure | There were no pre-defined cut-off scores for domains to be considered at the consensus meeting. At this meeting, participants agreed on the overall recommendations at the end of the Delphi process. | Unclear for acute pain. 6 domains for chronic pain | 6 domains for acute pain. 8 domains for chronic pain |
|  | **Was Delphi the final method for reaching consensus, or was it followed by another consensus process** | **Definition of consensus** | **Initial number of outcomes/ domains** | **Number of outcomes/ domains recommended** |
| **Miller [7]** | Results of Delphi process were used to help participants at subsequent meetings decide which outcomes to measure | There were no pre-defined cut-off scores for outcomes to be considered, at the consensus meeting, for inclusion in the core set. At this meeting, if at least 70% of participants felt an outcome should be measured in clinical research studies, it was included in the final core set | unclear | 5 domains, 7 outcomes |
| **Ruperto [32]** | Delphi process was followed by a meeting at which NGT was used to determine the final core set | Only outcomes suggested by at least 10 participants in Round 1 were carried forward to Round 2. There were no pre-defined cut-off scores for outcomes to be considered, at the consensus meeting, for inclusion in the core set. At this meeting, if at least 70% of participants felt an outcome should be measured in clinical research studies, it was included in the final core set | Unclear how many outcomes initially suggested. 41 outcomes for SLE _c_ and 37 for JDM were considered in Round 2 | SLE _b_: 8 domains, 11 outcomes. JDM: 11 domains, 15 outcomes |
| **Serrano [33]** | Delphi was used to rank the importance of outcomes, to help design relevant systematic reviews | Outcomes were ranked in order of importance after participants scored each from 1-10. | 11 outcomes | 11 outcomes |
| **Taylor 2005 [34]** | Aim of Delphi was not to identify a core set of outcomes, but to inform future consensus meetings | There was no pre-defined level of consensus about which domains should be measured. The authors did split the domains into ‘higher scoring’ and ‘lower scoring’ | 26 domains (spanning all situations) | 6 domains for rehabilitation trials, 7 domains for trials of disease controling drugs, 5 domains for trials of disease modifying drugs |
| **Taylor 2008 [35]** | Delphi was final method for reaching consensus | Outcomes were scored out of 7 as follows: 1-3=should definitely include in core set, 4=uncertain, 7-9=should definitely not include. In Round 2, all outcomes from Round 1 were presented again. In Round 3, only outcomes for which there was disagreement (ie bimodal response) or a median score of 4. After Round 3, outcomes with a median score of 1-3, and for which there was no disagreement, were included in the core set | 7 domains for acute gout, 15 domains for chronic gout | 6 domains for acute gout, 10 domains for chronic gout |
| **Zochling [36]** | Delphi was final method for reaching consensus _d_ | In the first two rounds, outcomes were removed if less than 80% of participants voted for its inclusion in the core set. In the final round, outcomes voted by at least 50% of participants were included in the core set | 7 domains | 7 domains |

Footnotes to Table 4:

a: The aim of the study was not to develop a core outcome set, but to provide a list of statements about specific outcomes in trials of certain therapies. For example, “the primary outcome measure of a reflux chest pain syndrome trial should be a clinically meaningful reduction of chest pain”.

b: The aim of the study was not to develop a core outcome set, but mainly to identify the most appropriate primary outcome, and to provide some guidance about the measurement and reporting of important secondary outcomes

c: SLE=Systemic Lupus Erythematosis; JDM=Juvenile Dermatomyositis

d: The primary aim of this study was to develop a list of core domains (called ‘elements’) that should be included in an international registry of patients with Ankylosing Spondylitis. 2/7 domains relate to demographic data and biologic-specific data, rather than outcomes of treatment. The remaining 5 domains relate to outcomes of treatment (clinical parameters, physical function, disease activity, imaging and Quality of Life)

References

1. Sinha IP, Williamson PR, Smyth RL (2009) Outcomes in Clinical Trials of Inhaled Corticosteroids for Children with Asthma Are Narrowly Focussed on Short Term Disease Activity. PLoS ONE 4: e6276.

2. Gandhi GY, Murad MH, Fujiyoshi A, Mullan RJ, Flynn DN, et al. (2008) Patient-Important Outcomes in Registered Diabetes Trials. JAMA 299: 2543-2549.

3. Guyatt G, Meade M (1997) Outcome measures: methodologic principles. Sepsis 1: 21-25.

4. Clarke M (2008) Standardising Outcomes in Paediatric Clinical Trials. PLoS Medicine 5.

5. Williamson PR, Gamble C, Altman DG, Hutton JL (2005) Outcome selection bias in meta-analysis. Statistical Methods in Medical Research 14: 515 - 524.

6. Dwan K, Altman DG, Arnaiz JA, Bloom J, Chan A-W, et al. (2008) Systematic review of the empirical evidence of study publication bias and outcome reporting bias. PLoS ONE 3: e3081.

7. Miller FW, Rider LG, Chung YL, Cooper R, Danko K, et al. (2001) Proposed preliminary core set measures for disease outcome assessment in adult and juvenile idiopathic inflammatory myopathies. Rheumatology 40: 1262-1273.

8. Clarke M (2007) Standardising outcomes for clinical trials and systematic reviews. Trials 8: 39.

9. Giannini EH, Ruperto N, Ravelli A, Lovell DJ, Felson DT, et al. (1997) Preliminary definition of improvement in juvenile arthritis.[see comment]. Arthritis & Rheumatism 40: 1202-1209.

10. Kirkham J, Dwan K, Altman D, Gamble C, Dodd S, et al. (2010) The impact of outcome reporting bias in randomised controlled trials on a cohort of systematic reviews. BMJ 340: c356.

11. Tugwell P, Boers M, Brooks P, Simon L, Strand V, et al. (2007) OMERACT: An international initiative to improve outcome measurement in rheumatology. Trials 8: 38.

12. Sinha I, Jones L, Smyth RL, Williamson PR (2008) A systematic review of studies that aim to determine which outcomes to measure in clinical trials in children. PLoS Medicine 5: e96.

13. Dalkey N, Helmer O (1963) An experimental application of the Delphi method to the use of experts. Management Science 9: 458-467.

14. Alahlafi A, Burge S (2005) What should undergraduate medical students know about psoriasis? Involving patients in curriculum development: modified Delphi technique. British Medical Journal 330: 633.

15. Morita T, Bito S, Kurihara Y, Uchitomi Y (2005) Development of a clinical guideline for palliative sedation therapy using the Delphi method. Journal of Palliative Medicine 8: 716-729.

16. Kellum J, Mehta R, Levin A, Molitoris B, Warnock D, et al. (2008) Development of a clinical research agenda for acute kidney injury using an international, interdisciplinary, three-step modified Delphi process. Clinical Journal of the American Society of Nephrology 3: 887.

17. Murphy M, Black N, Lamping D, McKee C, Sanderson C, et al. (1998) Consensus development methods, and their use in clinical guideleine development. Health technology assessment 2.

18. Brunner F, Lienhardt SB, Kissling RO, Bachmann LM, Weber U (2008) Diagnostic criteria and follow-up parameters in complex regional pain syndrome type I–a Delphi survey. European Journal of Pain 12: 48-52.

19. Jones J, Brown E, Volicer L (2000) Target outcomes for long term oral health care in dementia: A Delphi approach. Journal of Public Health Dentistry 60: 330-334.

20. van Hulst LTC, Fransen J, den Broeder AA, Grol R, van Riel PLCM, et al. (2009) Development of quality indicators for monitoring of the disease course in rheumatoid arthritis. Annals of the Rheumatic Diseases 68: 1805-1810.

21. Weigl M, Cieza A, Andersen C, Kollerits B, Amann E, et al. (2004) Identification of relevant ICF categories in patients with chronic health conditions: a Delphi exercise. Journal of Rehabilitation Medicine 36: 12 - 21.

22. Radtke MA, Reich K, Blome C, Kopp I, Rustenbach SJ, et al. (2009) Evaluation of quality of care and guideline-compliant treatment in psoriasis. Development of a new system of quality indicators. Dermatology 219: 54-58.

23. Dent J, Kahrilas PJ, Vakil N, Van Zanten SV, Bytzer P, et al. (2008) Clinical trial design in adult reflux disease: a methodological workshop. Alimentary Pharmacology & Therapeutics 28: 107.

24. Devane D, Begley CM, Clarke M, Horey D, O'Boyle C (2007) Evaluating maternity care: a core set of outcome measures. pp. 164 - 172.

25. Distler O, Behrens F, Pittrow D, Huscher D, Denton CP, et al. (2008) Defining appropriate outcome measures in pulmonary arterial hypertension related to systemic sclerosis: a Delphi consensus study with cluster analysis. Arthritis & Rheumatism 59: 867-875.

26. Douglas RS, Tsirbas A, Gordon M, Lee D, Khadavi N, et al. (2009) Development of criteria for evaluating clinical response in thyroid eye disease using a modified Delphi technique. Archives of Ophthalmology 127: 1155-1160.

27. Khan F (2007) Use of the International Classification of Functioning, Disability and Health (ICF) to identify preliminary comprehensive and brief core sets for multiple sclerosis. Disability & Rehabilitation 29: 205-213.

28. Khanna D, Lovell DJ, Giannini E, Clements PJ, Merkel PA, et al. (2008) Development of a provisional core set of response measures for clinical trials of systemic sclerosis. Annals of the Rheumatic Diseases 67: 703-709.

29. Lux AL, Osborne JP (2004) A proposal for case definitions and outcome measures in studies of infantile spasms and West syndrome: consensus statement of the West Delphi group. Epilepsia 45: 1416-1428.

30. McGrath PJ, Walco GA, Turk DC, Dworkin RH, Brown MT, et al. (2008) Core outcome domains and measures for pediatric acute and chronic/recurrent pain clinical trials: PedIMMPACT recommendations. Journal of Pain 9: 771-783.

31. Mease PJ, Arnold LM, Crofford LJ, Williams DA, Russell IJ, et al. (2008) Identifying the clinical domains of fibromyalgia: contributions from clinician and patient Delphi exercises. Arthritis & Rheumatism 59: 952-960.

32. Ruperto N, Ravelli A, Murray KJ, Lovell DJ, Andersson-Gare B, et al. (2003) Preliminary core sets of measures for disease activity and damage assessment in juvenile systemic lupus erythematosus and juvenile dermatomyositis. Arthritis care research 42: 1452-1459.

33. Serrano-Aguilar P, Trujillo-Martin MM, Ramos-Goni JM, Mahtani-Chugani V, Perestelo-Perez L, et al. (2009) Patient involvement in health research: a contribution to a systematic review on the effectiveness of treatments for degenerative ataxias. Social Science & Medicine 69: 920-925.

34. Taylor WJ (2005) Preliminary identification of core domains for outcome studies in psoriatic arthritis using Delphi methods. Ann Rheum Dis 64: ii110-112.

35. Taylor WJ, Schumacher HR, Jr., Baraf HS, Chapman P, Stamp L, et al. (2008) A modified Delphi exercise to determine the extent of consensus with OMERACT outcome domains for studies of acute and chronic gout. Annals of the Rheumatic Diseases 67: 888-891.

36. Zochling J, Sieper J, van der Heijde D, Braun J (2008) Development of a Core Set of Domains for Data Collection in Cohorts of Patients with Ankylosing Spondylitis Receiving Anti-Tumor Necrosis Factor-a Therapy. J Rheumatol 35: 1079-1082.

37. Oddis CV, Rider LG, Reed AM, Ruperto N, Brunner HI, et al. (2005) International consensus guidelines for trials of therapies in the idiopathic inflammatory myopathies. Arthritis & Rheumatism 52: 2607-2615.

38. Mease PJ, Clauw DJ, Arnold LM, Goldenberg DL, Witter J, et al. (2005) Fibromyalgia syndrome. Journal of Rheumatology 32: 2270-2277.

39. Carr A, Hewlett S, Hughes R, Mitchell H, Ryan S, et al. (2003) Rheumatology outcomes: the patient's perspective. Journal of Rheumatology 30: 880-883.

40. Kirwan J, Newman S, Tugwell P, Wells G (2009) Patient Perspective on Outcomes in Rheumatology—A Position Paper for OMERACT 9. The Journal of Rheumatology 36: 2067.

41. Goodman CM (1987) The Delphi technique: a critique. Journal of Advanced Nursing 12: 729-734.

42. Duncan PW, Jorgensen HS, Wade DT (2000) Outcome measures in acute stroke trials: a systematic review and some recommendations to improve practice.[see comment]. Stroke 31: 1429-1438.

43. Sackman H (1975) A Delphi Critique. Massachusetts: Lexington Books.

44. Bardecki M (1984) Participants' response to the Delphi method: An attitudinal perspective. Technological Forecasting and Social Change 25: 281-292.

**Financial disclosure**

Ian Sinha was funded by the NIHR Medicines for Children Research Network Clinical Trials Unit and Co-ordinating Centre. The Medicines for Children Research Network is part of the National Institute for Health Research (NIHR), and is funded by the Department of Health. The funders had no role in study design, data collection and analysis, decision to publish or preparation of the manuscript.

**Abbreviations**

JDM: Juvenile dermatomyositis; SLE: Systemic lupus erythematosis; NGT: Nominal group technique

PRISMA CHECKLIST

| **Section/topic** | **#** | **Checklist item** | **Reported on page #** |
| --- | --- | --- | --- |
| **TITLE** | | |  |
| Title | 1 | Identify the report as a systematic review, meta-analysis, or both. | 1 |
| **ABSTRACT** | | |  |
| Structured summary | 2 | Provide a structured summary including, as applicable: background; objectives; data sources; study eligibility criteria, participants, and interventions; study appraisal and synthesis methods; results; limitations; conclusions and implications of key findings; systematic review registration number. | 1 |
| **INTRODUCTION** | | |  |
| Rationale | 3 | Describe the rationale for the review in the context of what is already known. | 3 |
| Objectives | 4 | Provide an explicit statement of questions being addressed with reference to participants, interventions, comparisons, outcomes, and study design (PICOS). | 4 |
| **METHODS** | | |  |
| Protocol and registration | 5 | Indicate if a review protocol exists, if and where it can be accessed (e.g., Web address), and, if available, provide registration information including registration number. | n/a |
| Eligibility criteria | 6 | Specify study characteristics (e.g., PICOS, length of follow-up) and report characteristics (e.g., years considered, language, publication status) used as criteria for eligibility, giving rationale. | 5 |
| Information sources | 7 | Describe all information sources (e.g., databases with dates of coverage, contact with study authors to identify additional studies) in the search and date last searched. | 5 |
| Search | 8 | Present full electronic search strategy for at least one database, including any limits used, such that it could be repeated. | 5 |
| Study selection | 9 | State the process for selecting studies (i.e., screening, eligibility, included in systematic review, and, if applicable, included in the meta-analysis). | 7 |
| Data collection process | 10 | Describe method of data extraction from reports (e.g., piloted forms, independently, in duplicate) and any processes for obtaining and confirming data from investigators. | 6 |
| Data items | 11 | List and define all variables for which data were sought (e.g., PICOS, funding sources) and any assumptions and simplifications made. | 6 |
| Risk of bias in individual studies | 12 | Describe methods used for assessing risk of bias of individual studies (including specification of whether this was done at the study or outcome level), and how this information is to be used in any data synthesis. | 6 |
| Summary measures | 13 | State the principal summary measures (e.g., risk ratio, difference in means). | n/a |
| Synthesis of results | 14 | Describe the methods of handling data and combining results of studies, if done, including measures of consistency (e.g., I^2^) for each meta-analysis. | 7 |

Page 1 of 2

| **Section/topic** | **#** | **Checklist item** | **Reported on page #** |
| --- | --- | --- | --- |
| Risk of bias across studies | 15 | Specify any assessment of risk of bias that may affect the cumulative evidence (e.g., publication bias, selective reporting within studies). | n/a |
| Additional analyses | 16 | Describe methods of additional analyses (e.g., sensitivity or subgroup analyses, meta-regression), if done, indicating which were pre-specified. | n/a |
| **RESULTS** | | |  |
| Study selection | 17 | Give numbers of studies screened, assessed for eligibility, and included in the review, with reasons for exclusions at each stage, ideally with a flow diagram. | 7 |
| Study characteristics | 18 | For each study, present characteristics for which data were extracted (e.g., study size, PICOS, follow-up period) and provide the citations. | 7 |
| Risk of bias within studies | 19 | Present data on risk of bias of each study and, if available, any outcome level assessment (see item 12). | n/a |
| Results of individual studies | 20 | For all outcomes considered (benefits or harms), present, for each study: (a) simple summary data for each intervention group (b) effect estimates and confidence intervals, ideally with a forest plot. | n/a |
| Synthesis of results | 21 | Present results of each meta-analysis done, including confidence intervals and measures of consistency. | n/a |
| Risk of bias across studies | 22 | Present results of any assessment of risk of bias across studies (see Item 15). | n/a |
| Additional analysis | 23 | Give results of additional analyses, if done (e.g., sensitivity or subgroup analyses, meta-regression [see Item 16]). | n/a |
| **DISCUSSION** | | |  |
| Summary of evidence | 24 | Summarize the main findings including the strength of evidence for each main outcome; consider their relevance to key groups (e.g., healthcare providers, users, and policy makers). | 13 |
| Limitations | 25 | Discuss limitations at study and outcome level (e.g., risk of bias), and at review-level (e.g., incomplete retrieval of identified research, reporting bias). | 14 |
| Conclusions | 26 | Provide a general interpretation of the results in the context of other evidence, and implications for future research. | 15 |
| **FUNDING** | | |  |
| Funding | 27 | Describe sources of funding for the systematic review and other support (e.g., supply of data); role of funders for the systematic review. | 31 |

*From:*  Moher D, Liberati A, Tetzlaff J, Altman DG, The PRISMA Group (2009). Preferred Reporting Items for Systematic Reviews and Meta-Analyses: The PRISMA Statement. PLoS Med 6(6): e1000097. doi:10.1371/journal.pmed1000097

For more information, visit: **www.prisma-statement.org**.

Page 2 of 2
